# Foreign body in esophagus: Case report

**DOI:** 10.1016/j.ijscr.2021.106417

**Published:** 2021-09-16

**Authors:** Bustamante M. Mauricio E, Maciel U. Javier A, Hernández G. Ana K, Rangel L. Goretti, Ramírez G. Luis R., Del Valle Díaz de León Rodrigo Alexis

**Affiliations:** a IMSS Centro Médico Nacional de Occidente, Mexico; b Hospital Civil de Guadalajara “Juan I. Menchaca”, Mexico; c ISSSTE Hospital Regional de León, Mexico; d Universidad Nacional Autonóma de México, Mexico

**Keywords:** Foreign body, Endoscopy, Esophagus

## Abstract

**Introduction and importance:**

The ingestion of foreign bodies is a frequent cause of consultation in the emergency department, especially in pediatric and elderly patients.

**Case presentation:**

We present the case of a 48-year-old male patient who arrived to the emergency department with dysphagia after food intake. The diagnosis is confirmed by simple neck tomography. After a failed endoscopy, he underwent surgery, with subsequent resolution of the condition.

**Discussion:**

The diagnosis is based on the clinical history, physical examination and is supported by extension studies such as radiography, tomography and/or endoscopy, this last one being also therapeutic.

**Conclusion:**

Although in most cases there is a spontaneous passage through the gastrointestinal tract, there is the possibility of requiring endoscopy (reported success greater than 95% of cases) or surgical treatment.

## 1 Introduction

The ingestion of a foreign body in the emergency department is more frequent than one might expect; predominantly in the pediatric population and in the elderly, with an incidence of 4%, associated with a mortality of 1500 patients per year in the United States [1]; in the young adult population, this condition is mostly associated with mental illness. Of all patients, about 10 to 20% require medical intervention [1].

The diagnosis is made clinically and is complemented with imaging studies, being simple X-ray the first choice, and in case of not achieving a definitive diagnosis, a tomographic study or endoscopy is indicated. The esophageal site of occlusion depends on different factors, which include: anatomical (areas of less light such as the upper third of the esophagus), associated pathology (cancer, sclerotherapy, etc.) and the nature of the foreign body (sharp, spherical, etc.) [2]. We report a case of a male with a foreign body in the esophagus, this work has been reported in line with the SCARE 2020 criteria [3].

## 2 Case report

This is a 48-year-old male patient, originally and resident of Guadalajara, México; married, christian, complete junior high, employed in an oil store. With a significant history of smoking from 15 to 28 years of age at a rate of 10 cigarettes (IT 6.5 packs/year), occasional alcohol consumption. With type 2 Diabetes Mellitus of 14 years of diagnosis on treatment with metformin 850 mg every 8 h, without other comorbidities.

**Image 1:**
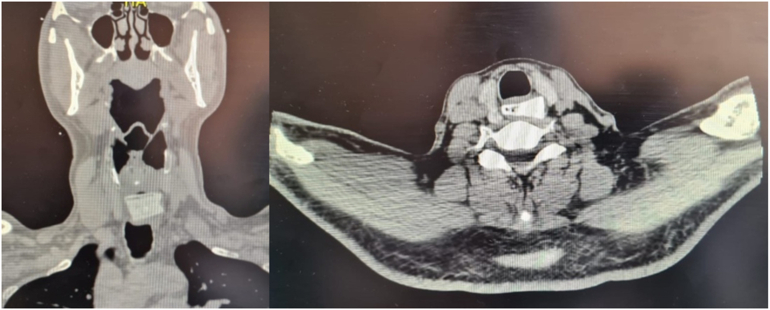
Simple coronal cut neck CT and Simple axial cut neck CT.

We present a clinical image consisting of a foreign body sensation in the esophagus after food intake, with dysphagia to liquids and solids, denying respiratory symptoms, attending the emergency department being evaluated by the Otorhinolaryngology service, performing laryngoscopy without evidence of a foreign body, therefore General Surgery service was consulted; a simple CT scan of the neck was performed, where the presence of a foreign body in the upper third of the esophagus of 20 × 25 mm was evidenced without data suggestive of perforation (Image 1).

**Image 2:**
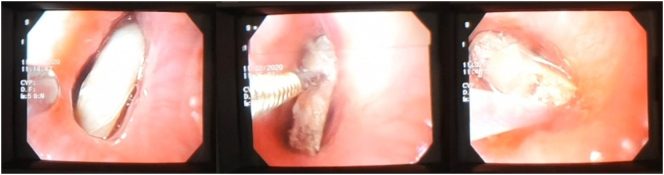
Foreign body in proximal esophagus, failed extraction with foreign body forceps and Failed Endoloop Extraction of Foreign Body.

The patient then is taken to Endoscopy service where an endoscopic study is performed with evidence of a foreign body, and failed extraction after multiple attempts with Endoloop and forceps (Image 2).

**Image 3:**
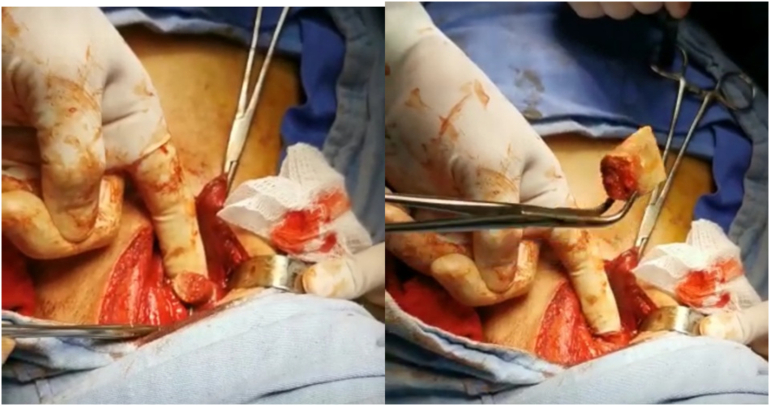
Evidence of a foreign body in the esophagus and foreign body removal.

After endoscopy treatment failure, surgical extraction was attempted, performing the procedure through a left lateral cervicotomy, and finding the foreign body in the cervical portion of the esophagus, which was extracted (Image 3) and subsequently closed esophagotomy. A General surgeon, with laparoscopic training and 20-year experience performed the procedure.

After the surgical procedure, the patient goes to standard floor hospitalization, where he presents a favorable evolution, however, occasional dysphonia and discharge from the surgical wound are evidenced. A new laryngoscopy is performed with evidence of left vocal cord paralysis and esophagogram, where contrast leakage is evidenced towards right bronchus, without extravasation of the contrast in the neck. In addition, a control endoscopy is performed, without evidence of fistula in the surgical area, for all the above, it is decided to give conservative management, with enteral nutrition by nasojejunal tube and wound care, as surgical wound infection was evidenced. Afterwards, with twice daily wound cleaning and antibiotic management, presents favorable evolution, discharging after 18 days of hospitalization. The patient was satisfied, the risks were previously explained to him and the final clinical evolution was favorable, without sequelae.

## 3 Discussion

Despite the fact that the ingestion of a foreign body is a frequent cause of consultation in the emergency room, in a third level institution like ours is not common to see this type of pathologies, since most of them are resolved at levels of less complexity with an endoscopy service. Our case is about an adult patient not in the population group age predominantly seen in this pathology, and without psychological pathology mostly seen in adults with this emergency.

The diagnosis was made clinically and is complemented with imaging studies, being simple radiography the first choice, which according to the European Society of Gastrointestinal Endoscopy is not necessary when the foreign body is not a bone or a spine and is not recommended perform tragus studies with contrast due to the risk of aspiration and hinder visibility during endoscopy [4]. In case of not achieving a definitive diagnosis, one can opt for a tomography (especially in suspicion of some complication) or endoscopy [4], in this case, this study method was carried out, due to the time of evolution, the facilities of the institution and the stability of the patient.

For the most part, cases of foreign body ingestion can be managed expectantly; there are factors that influence the decision of the treating physician [5], [6]. It is reported in a study that this pathology is the second cause of endoscopic urgency. Being frequent etiologies, food boluses, fish bones and as in the case of our patient, the ingestion of bones representing up to 4.34% of the cases. While there is a greater risk of impaction of objects in patients with pre-existing esophageal pathology, which in a study was found to be 5.5% [1], in this case, there was no evidence of esophageal pathology, which has been reported to be present in up to 62% of patients [6].

There are indications to carry out an urgent endoscopy within the first 6 h, in this clinical context the presence of a sharp object was found, while the other indications for urgent endoscopy are: complete obstruction of the esophagus and the ingestion of batteries. In the absence of this, the indication is to carry out endoscopy in the first 24 h, being the modality of choice the use of a flexible endoscope, as was our case described; although the complication rate does not vary compared to the use of a rigid endoscope [7].

Endoscopy has become the treatment of choice, since technological progress and staff training have evolved over time, with a success rate of up to 95.6%; this has generated a decrease in health costs and decreased the proportion of associated complications [2]. In the present case, this treatment was initially indicated; but it was unsuccessful despite multiple attempts.

In cases where it is not possible to solve the impaction via endoscopy, surgical treatment becomes relevant, as was the case presented; where in the literature, it is reported that up to 1.6% of patients warrant surgery, either due to failure of endoscopic treatment or due to the existence of an esophageal perforation [5], [7].

The postoperative evolution of the patient was satisfactory, without sequels. Due to the characteristics of the foreign body a complication like esophageal perforation was expected, but it resolved with conservative treatment.

## 4 Conclusion

Although the ingestion of foreign bodies is a condition most frequently seen in children, there is a considerable number of cases in the adult population, which is associated with psychiatric conditions or elderly. Deferring medical treatment can increase the risk of complications. As in this case, the failure in endoscopic management conditions the need to perform surgical treatment, which represents greater morbidity and mortality, that is why the management of these patients should be carried out in centers where the necessary resources, trained personnel and a multidisciplinary team can be offered.

## Ethical approval

The study is exempt from ethical approval.

## Consent

“Written informed consent was obtained from the patient for publication of this case report and accompanying images. A copy of the written consent is available for review by the Editor-in-Chief of this journal on request”.

## Guarantor

Luis Ricardo Ramírez González

## Provenance and peer review

Not commissioned, externally peer-reviewed.

## CRediT authorship contribution statement

Study concepts: Luis Ricardo Ramírez González.

Study design: Mauricio Eduardo Bustamante Morales.

Data acquisition: Javier Alejandro Maciel Urzúa.

Data analysis and interpretation: Ana Karen Hernández Guzmán

Manuscript preparation: Rodrigo Alexis del Valle Díaz de León.

Manuscript editing: Rodrigo Alexis del Valle Díaz de León.

Manuscript review: Goretti Rangel León

## Declaration of competing interest

The authors declare that the research was conducted in the absence of any commercial or financial relationships that could be construed as a potential conflict of interest.
